# Feasibility of implementing a standardized preeclampsia risk screening tool into the electronic medical record

**DOI:** 10.1002/pmf2.70205

**Published:** 2025-12-28

**Authors:** Daniella Rogerson, Carolina Thorlund‐Díaz, Marni B. Jacobs, Lillian Blank, Omar Mesina, Ayelet Ruppin‐Pham, Maryam Tarsa, Cynthia Gyamfi‐Bannerman, E. Nicole Teal

**Affiliations:** ^1^ Department of Obstetrics, Gynecology and Reproductive Sciences University of California San Diego La Jolla California USA; ^2^ San Diego School of Medicine University of California La Jolla California USA; ^3^ Department of Quality UC San Diego Health San Diego California USA; ^4^ Division of Maternal‐Fetal Medicine University of California San Diego La Jolla California USA

**Keywords:** electronic health record, low‐dose aspirin, preeclampsia, screening tool

## Abstract

**Background:**

Despite the fact that low‐dose aspirin (LDA) is the only effective intervention for preeclampsia risk reduction, not all those at increased risk of preeclampsia are recommended it. This study examines feasibility of implementing a preeclampsia risk screening tool into the electronic health record (EHR) to prompt LDA recommendation when appropriate and assesses whether there are differences in screening rates by race/ethnicity, language, or insurance payor.

**Methods:**

A standardized preeclampsia risk screening tool was launched in September 2022, which was intended to be used for all obstetrical patients to assess LDA eligibility. To assess feasibility of this tool, we conducted a retrospective cohort study of patients who delivered at an academic medical center from July 2023 to June 2024. The primary outcome of this was feasibility, defined as percentage of patients who delivered each month who had been screened using the tool. The secondary outcome was association between sociodemographic characteristics and screening. Chi‐square and Student's *t*‐tests were used to compare characteristics between screened status, and multivariable logistic regression determined strength of association. Logistic regression was used to determine differences in screening rates over each month.

**Results:**

A total of 4556 patients met inclusion criteria, and 2521 (55.3%) were screened using the tool. Tool use increased from 46.2% in the first month to 67.4% in the last month of the study, with the odds of the tool being used increasing on average 7% per month over 1 year (*p* < 0.05). Compared to patients who identify as Non‐Hispanic White, patients who identify as Hispanic/Latino had lower odds of being screened when adjusting for ethnicity/race, language, insurance, body mass index (BMI), and age (adjusted odds ration [aOR], 0.75; 95% confidence interval [CI], 0.62–0.89). In the same adjusted analysis, non‐English speaking patients were less likely to be screened than English‐speaking (aOR, 0.51; 95% CI, 0.36–0.71 and aOR, 0.51; 95% CI, 0.36–0.73) and publicly insured patients were less likely to be screened than privately insured (aOR, 0.83; 95% CI, 0.72–0.97).

**Conclusions:**

Preeclampsia risk screening increased substantially after implementing this standardized tool into the EHR which demonstrates its feasibility. We observed disparities in screening rates by race/ethnicity, language, and insurance payor.

## 1 BACKGROUND

Preeclampsia is a hypertensive disorder of pregnancy (HDP) that complicates 2%–8% of pregnancies globally and places the fetus at risk of intrauterine growth restriction and preterm birth, potentially complicated by long‐term sequelae [1, 2]. As such, it is estimated that preeclampsia is responsible for greater than 500,000 fetal and greater than 70,000 maternal deaths worldwide annually [3]. However, these numbers reflect significant disparities: in the United States, Black and American Indian/Alaska Native pregnant individuals account for nearly 21% and 17%, respectively, of all cases of HDP, including preeclampsia—compared to White individuals who account for 15% of cases [4]. Another report found that the rate of preeclampsia was 60% higher in Black patients than White patients [5]. Furthermore, between 2017 and 2019, HDP caused nearly 10% of deaths among Black individuals, while HDP associated deaths accounted for only 5% of deaths among White individuals [6]. This clear health inequity underscores the importance of evidence‐based interventions that can mitigate preeclampsia risk across historically marginalized populations.

One such intervention is low‐dose aspirin (LDA), which has been shown to reduce risk of preeclampsia and associated preterm birth by up to 10% and 24%, respectively [7, 8, 9]. It is also associated with decreased risk of preterm birth, fetal or neonatal death, small for gestational age infants, and composite serious adverse maternal and neonatal outcomes [7, 9, 10]. Accordingly, the American College of Obstetricians and Gynecologists (ACOG), the Society for Maternal‐Fetal Medicine, and US Preventive Services Task Force recommend nightly LDA initiated between 12 and 28 weeks of gestation for at‐risk pregnant individuals [10, 11, 12]. At‐risk patients are identified based on the ACOG criteria of having 1 high risk factor (history of preeclampsia, multifetal gestation, chronic hypertension, kidney disease, diabetes mellitus, and autoimmune conditions) or 2+ moderate risk factors (including but not limited to nulliparity, obesity, and low socioeconomic status) [11]. Despite these guidelines, studies have indicated that fewer than 50% of patients who meet criteria for LDA prescription for preeclampsia risk reduction receive a prescription or recommendation [13, 14]. There are limited data available as to whether there are sociodemographic differences in LDA prescription; however, some studies suggest there are disparities in LDA uptake and adherence, raising the question of disparities in LDA use [15].

Given the suboptimal rates of LDA prescription among at‐risk people, electronic healthcare record (EHR) tools have been developed across many institutions to help identify these patients and prompt prescription. Feasibility is a measure within the implementation science context that examines the extent to which a given intervention is of practical use within its assigned setting, usually measured by examining participation in the intervention under question [16]. This study aims to examine feasibility of incorporating a standardized preeclampsia risk screening tool into the EHR at our academic medical center, by evaluating its rate of utilization over time. The secondary aim is to examine sociodemographic factors associated with being screened for LDA eligibility with the goal of identifying disparities that arise upstream of subsequent preeclampsia outcomes.

## 2 METHODS

This is a retrospective cohort study of patients who delivered at a large, academic medical center from July 2023 to June 2024. The study was conducted to assess feasibility of integrating a standardized preeclampsia risk screening tool into the EHR. The screening tool was launched in September 2022; study dates were selected to exclude patients who delivered within 1 year of the screening tool launch date and thus could not have been screened. Inclusion criteria were research‐eligible patients in the UC San Diego Health System with admission and subsequent delivery during the study period.

**FIGURE 1:**
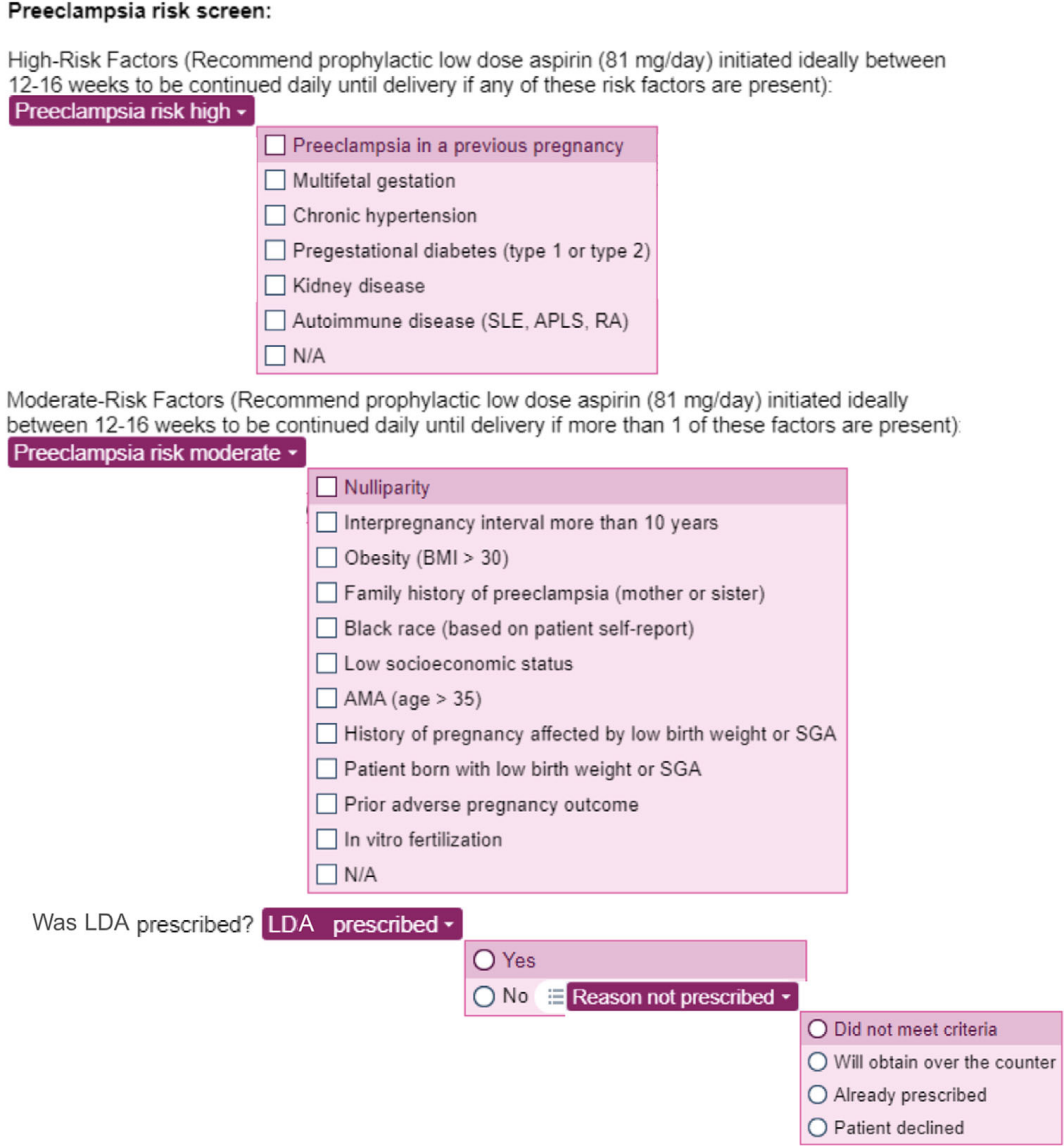
Interactive preeclampsia risk screening tool in the electronic medical record. AMA, advanced maternal age; APLS, antiphospholipid antibody syndrome; BMI, body mass index; LDA, low‐dose aspirin; N/A, not applicable; RA, rheumatoid arthritis; SGA, small for gestational age; SLE, systemic lupus erythematosus.

The screening tool was a standardized EHR template with discrete fillable fields that was created and used within the Epic EHR (Figure 1). The tool is an Epic SmartPhrase, which a provider can insert into the first prenatal visit note by typing a saved keyphrase. Once inserted into a clinical note by typing a period followed by the keyphrase, the interactive tool prompts providers to click through a series of dropdown menus, where they select any applicable high and moderate risk factors for preeclampsia. A dropdown menu then appears that prompts providers to prescribe LDA for patients who have at least one high risk factor or two moderate risk factors, in accordance with ACOG, the Society for Maternal‐Fetal Medicine, and the US Preventive Services Task Force guidelines [12, 17]. Lastly, the tool then prompts the provider to document whether LDA was prescribed, and if not prescribed, whether this was due to not meeting criteria, the patient obtaining the prescription over the counter, LDA already being prescribed, or the patient declining.

The tool was intended to be used for all obstetrical patients to determine LDA eligibility. It was available at all outpatient antenatal clinical sites and clinics where obstetrical patients are seen, and utilizing providers included physicians, resident physicians, certified nurse midwives, and advanced practice providers. Educational emails were sent during the first few months that the tool was available to inform providers of its availability, rational, and intended use. Whether or not this screening tool was utilized and other demographic factors were abstracted from the Epic Clinical Data Warehouse using SQL Server Management Studio (SSMS).

Patients were divided into two groups, those who were screened for preeclampsia risk and LDA eligibility with a completed EHR tool at any point in pregnancy, and those in whom screening was missed or not documented. The primary outcome was feasibility, defined as percentage of patients who delivered in the study period and had been screened during prenatal care using the standardized preeclampsia risk screening tool per month. This metric was selected as it reflects the percent of providers who participated in use of the LDA screening tool once it was made available over time. The secondary outcome was association between sociodemographic characteristics and screening rates. Race and ethnicity were self‐reported and listed in the demographic section of the Epic EHR. Language is self‐reported as the patient's primary preferred language; whether the patient spoke additional languages, including English, is not recorded. Chi‐square and Student's *t*‐tests were used to compare characteristics between screened status, and multivariable logistic regression determined strength of association in a model adjusted for significant differences in baseline demographics. Logistic regression was used to determine differences in screening rates over each month after 1 year of tool implementation. Statistical tests were performed using StataNow/BE 18.5. This study received approval from the institutional review board at our institution (IRB Number 810343); given the retrospective nature of the data in this study, patients were not required to provide consent.

## 3 RESULTS

**FIGURE 2:**
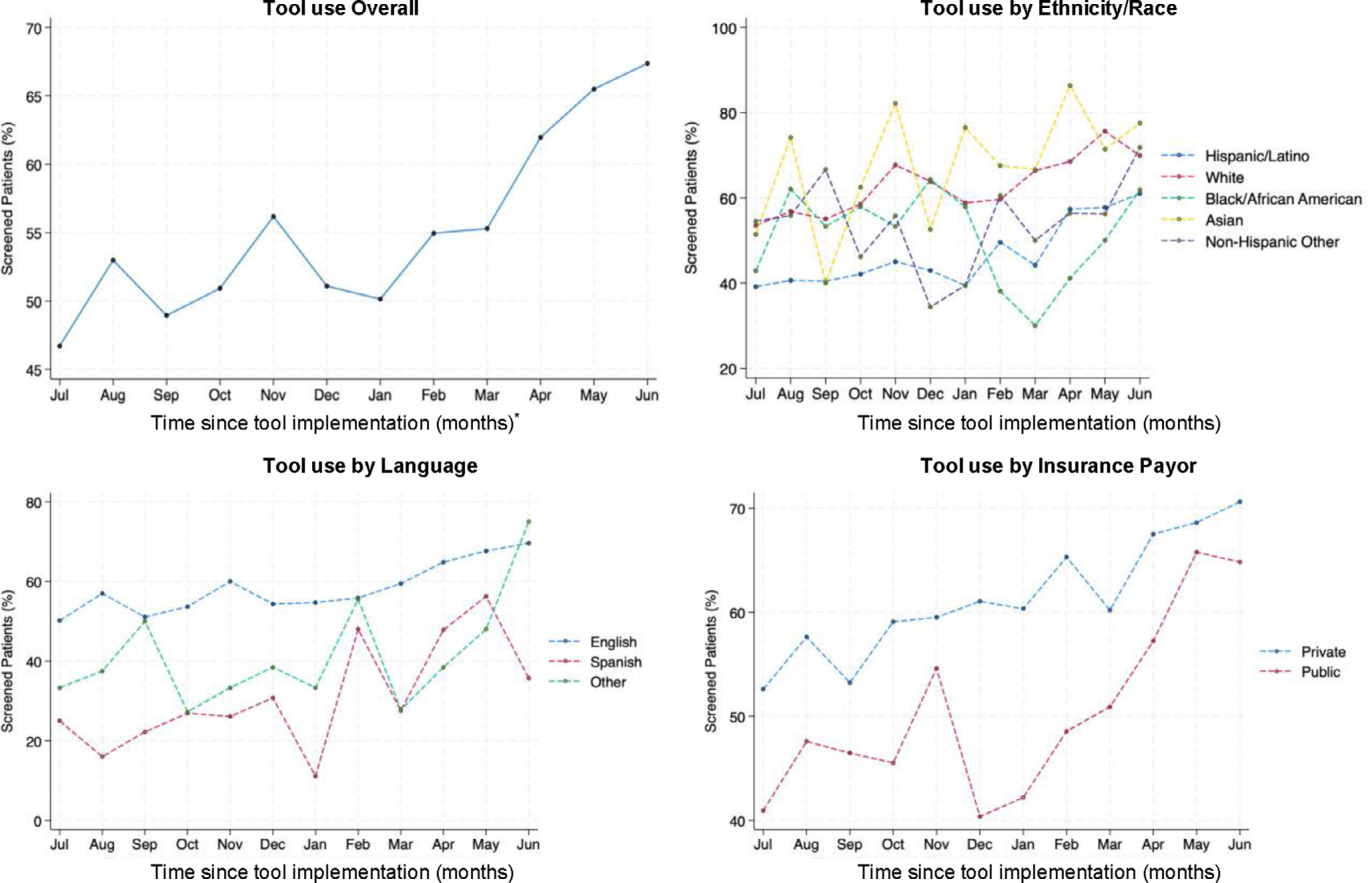
Screening tool use over time (percent) per month 1 year after implementation overall and by ethnicity/race, language, and insurance. ^*^ *p* < 0.05.

During the study period there were 4556 deliveries. Of those, 2521 (55.3%) were screened with the screening tool while 2035 (45.7%) were not. Baseline characteristics for the cohort are presented in Table 1. On average, patients who were not screened were younger and had higher body mass index (BMI) than patients who were screened (*p* < 0.001). Differences in ethnicity/race were noted, with more Hispanic/Latino patients in the not screened group (43.5% vs. 29.9%) (*p* < 0.001). Patients who were not screened were more likely to speak Spanish (10.7% vs. 3.9%) or another language (7.7% vs. 4.3%), and have public insurance (48.3% vs. 37.9%) than those who were screened (*p* < 0.001). The screening tool use rate increased from 46.2% in the first month to 67.4% in the last month of the study, and the odds of being screened increased 7% for each additional month after tool implementation (*p* < 0.05) (Figure 2).

**TABLE 1:** Participant characteristics by LDA screening tool use status.

Mean (SD) or *n* (%)	Screened *n* = 2521 (55.3%)	Not screened *n* = 2035 (44.7%)	*p* value
Maternal age	31.9 (5.2)	30.9 (6.3)	**<0.001**
Nulliparous			0.43
No	1384 (54.9%)	1141 (56.1%)	
Yes	1137 (45.1%)	894 (43.9%)	
BMI	27.7 (6.1)	29.1 (6.3)	**<0.001**
Ethnicity/race			**<0.001**
Hispanic/Latino	720 (29.9%)	825 (43.5%)	
Non‐Hispanic White	990 (41.1%)	580 (30.8%)	
Non‐Hispanic Black/African American	140 (5.8%)	131 (6.9%)	
Asian	304 (12.6%)	146 (7.7%)	
Non‐Hispanic Other	257 (10.7%)	216 (11.4%)	
Language			**<0.001**
English	2314 (91.8%)	1649 (81.6%)	
Spanish	99 (3.9%)	216 (10.7%)	
Other	108 (4.3%)	155 (7.7%)	
Insurance			**<0.001**
Private	1484 (62.1%)	938 (51.7%)	
Public	906 (37.9%)	877 (48.3%)	

*Note*: Categorical variables (nulliparous, ethnicity/race, language, and insurance) were compared using χ^2^. Continuous variables (maternal age and BMI) were compared using *t*‐test.

Bolded values are those that are statisically signifigant.

Abbreviations: BMI, body mass index; LDA, low‐dose aspirin; SD, standard deviation.

**FIGURE 3:**
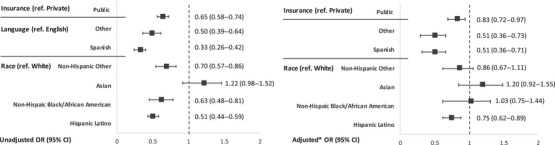
Multivariable analyses of sociodemographic factors associated with LDA screening tool use. ^*^ Model adjusted for ethnicity/race, language, insurance, BMI, and maternal age. BMI, body mass index; CI, confidence interval; LDA, low‐dose aspirin; OR, odds ratio.

In a multivariable analysis (Figure 3), patients who identified as Hispanic/Latino (odds ratio [OR], 0.51; 95% confidence interval [CI], 0.44–0.59), Non‐Hispanic Black/African American (OR, 0.63; 95% CI 0.48–0.81), or Other/Multiple race (OR, 0.70; 95% CI, 0.57–0.86) were less likely to be screened than patients who identified as Non‐Hispanic White. Patients who spoke Spanish (OR, 0.33; 95% CI, 0.26–0.42) or another language (OR, 0.50; 95% CI, 0.39–0.64) were less likely to be screened than patients who spoke English, and patients with public insurance were less likely to be screened than those with private insurance (OR, 0.65; 95% CI, 0.58–0.74). In a model adjusting for ethnicity/race, language, insurance, BMI, and maternal age (Figure 3), patients who identified as Hispanic/Latino had lower odds of being screened than patients who identified as Non‐Hispanic White (adjusted OR [aOR], 0.75; 95%, 0.62–0.89). Patients who spoke Spanish (aOR, 0.51; 95% CI, 0.36–0.71) or another language (aOR, 0.51; 95% CI, 0.36–0.73), were less likely to be screened than those who spoke English. Lastly, patients with public insurance were less likely to be screened with those than private insurance (aOR, 0.83; 95% CI, 0.72–0.97).

## 4 DISCUSSION

We found that implementation of a standardized preeclampsia risk screening tool to assess obstetrical patients for LDA eligibility at a large academic medical center was a feasible method of increasing assessment of preeclampsia risk, with the odds of the tool being used increasing on average 7% per month over 1 year. However, there were significant disparities in screening: patients who identified as Hispanic/Latino, did not speak English, or had public insurance were less likely to be screened for LDA eligibility than their counterparts who identified as Non‐Hispanic White, spoke English, and had public insurance.

Though use of this preeclampsia risk screening tool increased over time after implementation, overall use was only 67.4% in the final month of the study. This is in line with available data. Two recent retrospective studies found that only between 39% and 65% of eligible pregnant patients received an LDA prescription or counseling [14, 15]. Similarly, the prospective findings of Tita et al. highlighted that only 76% of patients with chronic hypertension (a high risk factor for preeclampsia) were taking LDA at the time of delivery [18]. Given the significant contribution of preeclampsia to maternal and neonatal morbidity and mortality in the United States and significant reduction in both preeclampsia and associated adverse outcomes with LDA use, these findings highlight the importance of expanded screening for LDA eligibility. It may be possible to increase use of the preeclampsia risk screening tool by making the optional Smart‐Phraase into a hard stop in the EHR, which requires completion in order to sign the encounter. Alternatively, this tool could be provided to the patient as a questionnaire (either paper or electronic) to be self‐completed at the new obstetrical visit, or filled out by trained medical assistants, in order to reduce provider burden while still ensuring adequate preeclampsia risk screening.

It is notable that screening for LDA eligibility was significantly lower among several populations previously highlighted to be among the highest risk for HDP and severe HDP. In 2014, the rate of preeclampsia was 70 and 47 per 1000 people among Black and Hispanic people compared to 43 per 1000 for White patients, and a larger proportion of cases were of severe preeclampsia among Black and Hispanic people (27 and 19 per 1000) than among White people (15 per 1000) [5]. Though low socioeconomic status and Black race are included as moderate risk factors for preeclampsia both by ACOG and within the preeclampsia risk screening tool at our institution, it is recognized that these are not risk factors in and of themselves but a proxy for systemic, institutional, and personal biases and health stressors that contribute to adverse pregnancy outcomes [17]. Our findings are relevant as they highlight disparities in upstream factors (screening for preeclampsia risk and LDA eligibility), which likely influence disparities downstream reflected in pregnancy outcomes (incidence, gestational age at onset, and severity of preeclampsia). Work targeted at identifying modifiable health system behaviors contributing to disparities in care is necessary, and future studies that assess the feasibility of tools and other methods to promote LDA prescription should examine disparities in use as well as overall application.

This study has limitations. Not all participants who delivered at our institution within the study period received prenatal care at our institution prior to 28 weeks’ gestation, and so may not have been screened with the preeclampsia risk tool. Use of the screening tool does not perfectly capture LDA prescription, as providers may prescribe aspirin without use of the tool. It is possible that many patients not screened with the tool did not receive LDA, but also possible that they were prescribed LDA without tool use. This may reflect patterns in LDA prescription which would not be captured by examining screening tool use. Additionally, this study did not capture pregnancy outcomes, thus we cannot comment on whether increased tool use over time or disparities in tool use influenced the incidence of preeclampsia. We were unable to stratify findings by clinical site or provider site, which may preclude detection of associations between these factors and LDA screening. Lastly, we were not able to collect survey data from providers about why they did or did not use the LDA screening tool, which prevents us from understanding barriers to use. We suspect the major barrier to tool use is limited provider time.

This study also has several strengths. Data from our large academic medical center with a diverse population are likely generalizable, and feasibility findings at this center are likely applicable to other large, academic medical centers with high overall volume and incidence HDP. Additionally, this study seeks to examine disparities in upstream factors that influence pregnancy outcomes and preeclampsia, and thus highlights actionable steps in addressing disparities in these outcomes.

## 5 CONCLUSIONS

Screening for LDA eligibility increased substantially after implementing a preeclampsia risk screening tool into the EHR, demonstrating its feasibility. However, despite this standardized screening tool, there were disparities in screening rates by race/ethnicity, language, and insurance payor. These data highlight the need for further studies examining disparities in upstream factors, which influence pregnancy outcomes including preeclampsia, preterm birth, and maternal and neonatal morbidity and mortality.

## CONFLICT OF INTEREST STATEMENT

The authors declare no conflicts of interest.

## ETHICS STATEMENT

Ethical approval for the study was obtained from the Institutional Review Board at UC San Diego, IRB Number 810343.

## Data Availability

The authors confirm that data supporting the findings of this study are available within the article.
